# Emergence of *Enterobacter cloacae* Complex Co-Producing IMP-10 and CTX-M, and *Klebsiella pneumoniae* Producing VIM-1 in Clinical Isolates in Japan

**DOI:** 10.3390/microorganisms8111816

**Published:** 2020-11-18

**Authors:** Satoshi Nishida, Naohisa Matsunaga, Yuta Kamimura, Shinobu Ishigaki, Taiji Furukawa, Yasuo Ono

**Affiliations:** 1 Department of Microbiology and Immunology, Teikyo University School of Medicine, 2-11-1 Kaga, Itabashi-ku, Tokyo 173-8605, Japan; onoy@med.teikyo-u.ac.jp; 2 Department of Infection Control and Prevention, Teikyo University Hospital, Itabashi, Tokyo 173-8605, Japan; oanslr@hotmail.com; 3 Department of Laboratory Medicine, Teikyo University Hospital, Itabashi, Tokyo 173-8605, Japan; yutakami@med.teikyo-u.ac.jp (Y.K.); ishigaki@med.teikyo-u.ac.jp (S.I.); tfrkw@med.teikyo-u.ac.jp (T.F.)

**Keywords:** carbapenemase, Enterobacteriaceae, IMP-1, metallo-β-lactamase, multidrug resistance, VIM-1

## Abstract

Background: Carbapenemase-producing Enterobacteriaceae (CPE) are an emerging threat in healthcare settings worldwide. Objectives: We evaluated the presence of carbapenemase genes in CPE in a tertiary care university hospital in Tokyo, Japan. Methods: Carbapenem-resistant clinical isolates were collected in 2018 at Teikyo University Hospital (Tokyo, Japan). Bacterial species were identified using MALDI-TOF MS. Carbapenemase production was evaluated using a carbapenemase inactivation method. The presence of carbapenemase genes was confirmed by multiplex PCR and DNA sequencing. Results: Four CPE isolates were identified: two *Enterobacter cloacae* complex strains and *Klebsiella oxytoca* and *Klebsiella pneumoniae* strains. Three of the isolates (*E. cloacae* complex and *K. oxytoca*) were IMP-1-type producers, including IMP-10 in their produced metallo-β-lactamase, and are epidemic in East Japan. The IMP-10-producing *E. cloacae* complex strain also produced CTX-M ESBL. The other CPE isolate (*K. pneumoniae*) is a VIM-1 producer. VIM-1-producing *K. pneumoniae* is epidemic in Europe, especially in Greece. Accordingly, the VIM-1 producer was isolated from a patient with a medical history in Greece. Conclusions: This study revealed the emergence of *E. cloacae* complex co-producing IMP-1-type carbapenemase and CTX-M ESBL, and *K. pneumoniae* producing VIM-1 carbapenemase in clinical isolates in Japan. Metallo-β-lactamase was the most prevalent type of carbapenemase at Teikyo University Hospital, especially IMP-1-type carbapenemase. The detection of VIM-1-producing *K. pneumoniae* suggests that epidemic CPE from overseas can spread to countries with low CPE prevalence, such as Japan, highlighting the need for active surveillance.

## 1. Introduction

Carbapenem-resistant Enterobacteriaceae (CRE) are often multidrug-resistant (MDR) and pose severe problems in terms of clinical treatment and infection control. Recently, carbapenemase production has become a common mechanism of carbapenem resistance in Enterobacteriaceae, which has given rise to carbapenemase-producing Enterobacteriaceae (CPE). The limited options for the treatment of MDR CPE infections significantly increases morbidity and mortality [1].

Carbapenemase-producing organisms (CPO), including CPE, are emerging worldwide and have caused many outbreaks. As an example, carbapenem-resistant *Acinetobacter baumannii* caused a massive outbreak at Teikyo University Hospital in 2009 [2]. Based on experience gained during these outbreaks and in line with the updated recommendations of the National Epidemiological Surveillance of Infectious Diseases for the control and prevention of healthcare-associated infections with CRE [3], Teikyo University Hospital implemented active surveillance and rapid feedback as infection control measures. The implementation of strict CPO screening resulted in the identification of CPOs in patients, especially in those with a medical history abroad, and in the enforcement of strict infection controls to prevent other outbreaks. For instance, an NDM-5-producing *Escherichia coli* was isolated from a patient from Bangladesh in Japan in 2013 [4]. An NDM-1-producing *Klebsiella pneumoniae* was isolated in 2014 from a patient with a medical history in Indonesia [4]. In 2016, multiple carbapenemase-producing Gram-negative bacilli were isolated from a single patient with an ICU admission history in Indonesia [5]. These CPOs were colistin-resistant KPC-2-producing *K. pneumoniae*, IMP-7-producing *Pseudomonas aeruginosa,* and OXA-23-producing *A. baumannii*. Recently, an OXA-48-like-producing *E. coli* was isolated from a patient treated with percutaneous transhepatic biliary drainage in India [6].

Here, we describe the results of a survey of carbapenemases among CPE collected at Teikyo University Hospital in 2018. In addition to admission screening, we screened inpatients for the presence of CPOs. We thus identified multiple metallo-β-lactamase (MBL)-producing Enterobacteriaceae. We report here the isolation of *Enterobacter cloacae* complex and *Klebsiella oxytoca* producing IMP-1, *Enterobacter cloacae* complex co-producing IMP-1-type MBL, and CTX-M ESBL from patients in Japan, and *K. pneumoniae* producing VIM-1 MBL from another patient, who had a medical history in Greece. Current and previous studies suggest that epidemic CRE has continuously spread to countries with low CRE prevalence, such as Japan. The present study stresses the importance of active antimicrobial resistance surveillance in hospital settings.

## 2. Materials and Methods

### 2.1. Isolation and Identification

Teikyo University Hospital has 1078 beds. Carbapenem-resistant Enterobacteriaceae were collected from January to December 2018. Seventy carbapenem-resistant clinical isolates were collected from 63,712 samples during 2018. Carbapenem resistance was defined as a minimum inhibitory concentration (MIC) ≥ 2 mg/L for imipenem. Microbiological analysis was performed as previously reported [5]. Briefly, faecal samples, tracheal samples (aspirate and sputum), and pharyngeal and wound swabs were collected for screening. To screen for ESBL-producing bacteria and CPOs, the samples were plated on CHROM agar ESBL/MDRA (Kanto Chemical, Tokyo, Japan), BTB agar (Eiken Chemical, Tokyo, Japan) with a piperacillin/tazobactam (100/10 μg) disc (Becton Dickinson, Franklin Lakes, NJ, USA), and NAC agar (Eiken Chemical) with an imipenem (10 μg) disc (Becton Dickinson). Antimicrobial susceptibility testing was performed using MicroScan WalkAway (Beckman Coulter, Brea, CA, USA). Antimicrobial susceptibility of isolates was defined according to the breakpoints listed in the CLSI guidelines M100-S24 [7]. Carbapenemase production was investigated using a carbapenemase inactivation method [8]. MBL production was confirmed by a double-disk synergy test using a sodium mercaptoacetic acid disc (Eiken Chemical) [9]. The isolates were identified using a MALDI biotyper (Bruker Daltonics, Billerica, MA, USA) and VITEK MS (bioMérieux, Marcy l’Etoile, France).

### 2.2. Multilocus Sequence Typing (MLST)

MLST was performed using the protocol developed by the Institut Pasteur (https://bigsdb.pasteur.fr) [10] and provided by PubMLST (https://pubmlst.org) [11,12]. Genomic DNA was isolated using a DNeasy Blood & Tissue kit (Qiagen, Hilden, Germany). DNA fragments were amplified by PCR using KOD FX Neo (Toyobo, Osaka, Japan) and specific primers (Supplemental Material) in a final volume of 50 µL. The amplification began with an initial denaturation at 94 °C for 2 min followed by 35 cycles of amplification using a step program (10 s at 98 °C, 30 s at the melting temperature (*T_m_*), and 1 min at 68 °C). The PCR products were treated with ExoSAP-IT Express (Affymetrix, Santa Clara, CA, USA) and directly sequenced as described in our previous report [2].

### 2.3. Resistance Gene Typing and Plasmid Replicon Typing

The Cica Geneus genotype detection kit (Kanto Chemical) was used for multiplex PCR to detect the ESBL (SHV, TEM, CTX-M-1, CTX-M-2, CTX-M-8, and CTX-M-9 groups) and carbapenemase (GES, IMP-1 group, IMP-6, KPC, NDM, OXA-48-like, and VIM) genes. Plasmid replicons were detected by PCR using KOD FX Neo (Toyobo) and specific primers (Supplemental Materials) [13]. DNA fragments containing MBL genes were amplified by PCR using KOD FX Neo (Toyobo) and specific primers (Supplemental Material) [14], treated with ExoSAP-IT Express (Affymetrix), and directly sequenced [2].

## 3. Results and Discussion

We collected 70 carbapenem-resistant clinical isolates during 2018. Among them, we identified four CPE as MBL-producing Enterobacteriaceae: two *E. cloacae* complex isolates, and *Klebsiella oxytoca* and *K. pneumoniae* isolates. CRE accounted for <0.2% of the bacterial isolations, and CPE accounted for <6% of the CRE infections, consistent with the results of a previous national surveillance report in 2014 [3]. The antimicrobial susceptibility profiles of the MBL-producing Enterobacteriaceae isolates, strain numbers, sample characteristics, MLST, carbapenemase production, and ESBL are summarised in Table 1. All the MBL-producing Enterobacteriaceae isolates were MDR.

The carbapenemase and ESBL genes harboured by the isolates were identified by multiplex PCR. Strain TK1601 harboured *bla*_IMP-1_; strain TK1602 harboured *bla*_VIM_ and *bla*_SHV_; strain TK1603 harboured *bla*_IMP-1_, *bla*_CTX-M-1_, and *bla*_TEM_; and strain TK1604 harboured *bla*_IMP-1_. Sequencing analysis identified the *bla*_VIM_ gene in *K. pneumoniae* and *bla*_IMP-1_ gene in an isolate of *E. cloacae* complex as *bla*_VIM-1_ and *bla*_IMP-10_, respectively.

The four Enterobacteriaceae isolates produced MBLs representing IMP-1-type and VIM-1 enzymes. IMP-1 is an epidemic MBL in Japan [15]. The distribution of IMP-producing Gram-negative bacteria in Japan is biased, such that, as reported, IMP-1 occurs more frequently in eastern Japan and IMP-6 occurs more frequently in western Japan [15,16]. The detection of IMP-1 at Teikyo University Hospital was consistent with such an IMP distribution in Japan. The detection of IMP-1-producing *K. oxytoca* at the University of Tokyo Hospital was previously reported in 2009 [17]. The detected strain may be similar to the one detected in the current study, as their drug sensitivity patterns, as reported, are identical (Table 2). The MLST of previous *K. oxytoca* was not performed. In this study, IMP-1-producing *K. oxytoca* and IMP-1-producing *E. cloacae* complex were found to belong to ST88 and ST252, respectively. *E. cloacae* complex ST252 was initially isolated as a KPC-3 producer from a liver-transplant patient in a hospital (PA, USA) in 2009 [18]. *E. cloacae* complex ST252 producing GES-5 was isolated from the leg wounds of a diabetic patient in a Czech hospital in 2016 [19]. IMP-1-producing *E. cloacae* ST252 and IMP-1-producing *K. oxytoca* ST88, such as the isolate characterised in this study, have never been reported. Therefore, IMP-1-producing *E. cloacae* ST252 and IMP-1-producing *K. oxytoca* ST88 might have emerged locally in Japan. This finding suggests that *E. cloacae* ST252 is an international clone of a carbapenemase reservoir, although this will require further investigation. Furthermore, in Tokyo, the in-hospital mortality of patients colonised by IMP-1-producing *E. cloacae* complex is high [20]. Therefore, it is necessary to continue to screen hospitalised patients for the presence of IMP-1-producing *E. cloacae* complex as an essential measure for infection control.

On the other hand, the current study identified an *E. cloacae* complex isolate co-producing IMP-10 and CTX-M-1 group enzymes. Of note, IMP-1-producing *E. cloacae* complex ST78 is a successful (prevalent and persistent) clone in Tokyo [15]. Interestingly, we identified IMP-10-producing *E. cloacae* complex ST78, suggesting a selection of an ST78 strain variant producing IMP-1 with a V49F (145 G to T) substitution [21,22]. The distribution of IMP-1 and IMP-10 enzymes was reported in other CPE species, such as *Serratia marcescens* in Tokyo [23]. IMP-1- and IMP-10-producing *S. marcescens* have Class I integrons similar to In*316* located in the plasmid pMTY11043_IncHI2 harboured by IMP-1-producing *E. cloacae* complex ST78 isolate TUM11043 [15,23]. Therefore, plasmids carrying Class I integrons encoding IMP-1 and IMP-10 might be widely distributed in CPE in Tokyo. Because IMP-10-producing *P. aeruginosa* was initially reported in Japan and Class I integrons encoding IMP-10 in *P. aeruginosa* isolates have a similar structure to the Class 1 integron in *S. marcescens*, the mobile genetic element carrying *bla*_IMP-10_ might have been transferred initially from *P. aeruginosa* to Enterobacteriaceae in Japan [21,23,24,25]. The MBL-producing Enterobacteriaceae isolates in this study share the incompatibility plasmids IncFIB and IncHI2. The IMP-10-producing *E. cloacae* complex and VIM-1-producing *K. pneumoniae* harboured the unique incompatibility plasmids IncL/M and IncHI1, respectively.

We isolated VIM-1-producing *K. pneumoniae* from a patient who had been previously hospitalised in Greece. Hospitalisation in Greece might be a risk factor for infection or colonisation by VIM-producing Enterobacteriaceae. For instance, an outbreak of VIM-1-producing *K. pneumoniae* from Greece in a hospital in France was reported in 2006 [26]. Many VIM-producing *K. pneumoniae* strains are being isolated in Greece [27]. The dominant clone of VIM-1-producing *K. pneumoniae* belongs to ST147 [28]. *K. pneumoniae* ST70 was characterised as an NDM-1 producer in Greece [29]. However, a VIM-1-producing *K. pneumoniae* ST70, such as the isolate characterised in the current study, has never been reported. The emerging clone of *K. pneumoniae* ST70 may have evolved to host a mobile genetic element encoding VIM-1.

VIM-2-producing *P. aeruginosa* has spread throughout Asia, including Japan [30]. By contrast, the VIM-producing Enterobacteriaceae isolate from Japan was reported as a VIM-2-producing *E. cloacae*, isolated from a patient in a paediatric ward [31]. However, the most notorious nosocomial pathogen, *K. pneumoniae*, producing VIM-1 has not yet been reported in Japan. VIM-2 is a significant epidemic carbapenemase harboured by *P. aeruginosa* in Japan. Therefore, the possibility that the VIM-1-encoding plasmid might be transferred from *P. aeruginosa* to *K. pneumoniae* in Japan may be excluded. On the other hand, VIM-1-producing *K. pneumoniae* is epidemic in Europe [1].

The findings presented herein suggest that a medical history in Greece might be linked to the CPE colonisation described in this study. This should serve as a warning about the possibility that doctors and other medical staff can come into contact with CPE when admitting patients colonised by CPE, and then spread the infection to other inpatients at the hospital. To reduce the spread of CPE in low-prevalence countries, such as Japan, the screening of all patients, especially those with a history of hospitalisation and travel abroad, is inevitable.

Given the limitations imposed by this surveillance study, the carbapenemase production by CRE may have been underestimated because of the relatively short screening period and the exclusion of CPE isolates but not resistant isolates. Although we detected MBL as a major carbapenemase in a CRE at Teikyo University Hospital in 2018, it strongly depended on the yearly screened patients (Table 3). The screening methods used to identify CPE also affected the results. Nevertheless, the implementation of active surveillance has helped to prevent the spread of CPO in the last decade at Teikyo University Hospital. Improvements in the methods for screening CPE may help to more precisely reveal the status of CPE colonization or infection in healthcare settings in the future.

In conclusion, we report here the isolation of *E. cloacae* complex co-producing IMP-1-type MBL and CTX-M ESBL, *E. cloacae* complex producing IMP-1 MBL, *K. oxytoca* producing IMP-1 MBL, and *K. pneumoniae* producing VIM-1 MBL in Japan. This study contributes to the delineation of recent epidemic trends and treatment options for CPE in Tokyo, Japan, and highlights the possibility of resistance gene transfer among Enterobacteriaceae as well as resistance gene spread from travellers returning from abroad. The study also highlights the need for active surveillance and analysis of the medical history of patients returning from travel abroad.

## Figures and Tables

**Table 1:** Antimicrobial susceptibility profiles of metallo-β-lactamase (MBL)-producing Enterobacteriaceae.

Patient	1	2	3	4
Sex	F	M	F	M
Age (years)	60	34	84	70
Date of isolation	7/5/2018	25/7/2018	4/9/2018	16/12/2018
Sample	Pharyngeal mucus	Faeces	Urine	Faeces
Species	*E. cloacae*	*K. pneumoniae*	*E. cloacae*	*K. oxytoca*
Strain number	TK1601	TK1602	TK1603	TK1604
MLST	ST252	ST70	ST78	ST88
Plasmid Inc	FIB, HI2	FIB, HI1	FIB, HI2, L/M	FIB, HI2
Carbapenemase	IMP-1	VIM-1	IMP-10	IMP-1
ESBL	–	–	CTX-M-1	–
Antimicrobial agent	**MIC (mg/L)** *^a^*			
Penicillins				
Ampicillin	**>16**	**>16**	**>16**	**>16**
Piperacillin	**>64**	**>64**	**>64**	**64**
Sulbactam/ampicillin	**>16**	**>16**	**>16**	**>16**
Amoxicillin/clavulanic acid	**>16**	**>16**	**>16**	**>16**
Tazobactam/piperacillin	**>64**	**>64**	**>64**	8
Cephalosporins				
Cefazolin	**>16**	**>16**	**>16**	**>16**
Cefotiam	**>16**	**>16**	**>16**	**>16**
Cefotaxim	**>2**	**>2**	**>2**	**>2**
Ceftazidime	**>16**	**>16**	**>16**	**>16**
Ceftriaxone	**>2**	**>2**	**>2**	**>2**
Cefepime	**>16**	**16**	**>16**	**16**
Cefozopran	**>16**	**>16**	**>16**	**8**
Cefmetazole	**>32**	**>32**	**>32**	**>32**
Cefaclor	**>16**	**>16**	**>16**	**>16**
Cefdinir	**>2**	**>2**	**>2**	**>2**
Cefpodoxime	**>4**	**>4**	**>4**	**>4**
Cefcapene	**>2**	**>2**	**>2**	**>2**
Flomoxef	**>32**	**>32**	**>32**	**32**
Sulbactam/cephoperazon	**>32**	**>32**	**>32**	**>32**
Carbapenems				
Doripenem	**4**	**8**	**>8**	**>8**
Imipenem	**4**	**4**	**>8**	**4**
Meropenem	**4**	**4**	**>8**	**8**
Monobactam				
Aztreonam	4	**8**	**>16**	4
Fluoroquinolones				
Ciprofloxacin	0.25	0.25	**>2**	1
Levofloxacin	0.5	0.5	**>4**	2
Sitafloxacin	1	1	**>2**	1
Aminoglycosides				
Gentamicin	2	**8**	**8**	2
Tobramycin	**8**	**>8**	**8**	4
Amikacin	4	8	4	4
Tetracycline Minocycline	**>8**	2	**>8**	**8**
Polymyxin				
Colistin	NT	2	NT	2
Other				
Fosfomycin	**16**	**>16**	**>16**	**16**
Trimethoprim/sulfamethoxazole	2	**>2**	**>2**	2

*^a^* Values in bold indicate resistant; NT, not tested.

**Table 2:** Antimicrobial susceptibility profiles of IMP-1-producing *K. oxytoca* from this study and a previous study [17].

Strain Number	TK1604	K27
Year of isolation	2018	2006
MLST	ST88	NT
Carbapenemase	IMP-1	IMP-1
ESBL	–	–
Cephalosporins		
Cefazolin	**>16**	**>128**
Cefotaxim	**>2**	**32**
Ceftazidime	**>16**	**>64**
Cefoperazone	NT	**>128**
Sulbactam/cefoperazone	**>32**	NT
Carbapenems		
Imipenem	**4**	**2**
Meropenem	**8**	**4**
Monobactam		
Aztreonam	4	1
Fluoroquinolones		
Ciprofloxacin	1	1
Levofloxacin	2	2
Aminoglycosides		
Gentamicin	2	1
Amikacin	4	2

Minimum inhibitory concentration (MIC) values in bold indicate resistant; NT, not tested.

**Table 3:** Carbapenemase-producing Gram-negative organisms isolated at Teikyo University Hospital, Tokyo, Japan.

Organism	Carbapenemase	MLST	Year of Isolation	Clinical History Location	Other Properties	Reference
*A. baumannii*	OXA-51-like		2009/2010	NA	MDR, outbreak	[2]
*E. coli*	NDM-5	ST540	2013	Bangladesh	NDM-5 in Japan	[4]
*K. pneumoniae*	NDM-1	ST76	2014	Indonesia	NDM-1 in Japan	[4]
*K. pneumoniae*	KPC-2	ST11	2016	Indonesia	PDR	[5,32]
*P. aeruginosa*	IMP-7	ST357	2016	Indonesia	XDR	[5]
*A. baumannii*	OXA-23, OXA-66	ST1050	2016	Indonesia	XDR	[5]
*E. coli*	OXA-48-like		2018	India	MDR	[6]
*K. pneumoniae*	VIM-1	ST70	2018	Greece	VIM-1 in Japan	This study
*K. oxytoca*	IMP-1	ST88	2018	NA	Epidemic in Japan	This study
*E. cloacae*	IMP-1	ST252	2018	NA	Epidemic in Japan	This study
*E. cloacae*	IMP-10	ST78	2018	NA	IMP-10 in Japan	This study

NA, not available; MDR, multidrug-resistant; PDR, pandrug-resistant; XDR, extensively drug-resistant.

## Supplementary Materials

The following are available online at https://www.mdpi.com/2076-2607/8/11/1816/s1.
